# Duplicated Genes on Homologous Chromosomes Decipher the Dominant Epistasis of the Fiberless Mutant in Cotton

**DOI:** 10.3390/biology14080983

**Published:** 2025-08-02

**Authors:** Yu Le, Xingchen Xiong, Zhiyong Xu, Meilin Chen, Yuanxue Li, Chao Fu, Chunyuan You, Zhongxu Lin

**Affiliations:** 1 National Key Laboratory of Crop Genetic Improvement, College of Plant Science and Technology, Huazhong Agricultural University, Wuhan 430070, China; leyu_hzau@163.com (Y.L.); xcxiong2016@163.com (X.X.); xuzhiyong@webmail.hzau.edu.cn (Z.X.); chenmeilin0623@163.com (M.C.); liyx1124@163.com (Y.L.); fuchao_666@webmail.hzau.edu.cn (C.F.); 2 Cotton Research Institute, Shihezi Academy of Agriculture Science, Shihezi 832011, China; 3 Xinjiang Uygur Autonomous Region Academy of Agricultural Sciences, Urumqi 830091, China

**Keywords:** cotton, fiber initiation, *GhMYB25like*, dominant epistasis, RNA-seq

## Abstract

Cotton fiber initiation determines the fiber yield, yet the genetic basis underlying lint and fuzz initiation has still not been fully uncovered. Here, map-based cloning was carried out to identify the fiberless mutant genes derived from a cross between *Gossypium hirsutum* acc. WT and a natural fiberless mutant, *fbl_SHZ_*. The 12:3:1 segregation ratio in F_2_ populations (including 1848 and 3100 individuals that were developed in 2016 and 2018, respectively) revealed dominant epistasis, with the fuzz gene exerting dominance over the lint gene. Genetic linkage analysis revealed that *GhMYB25like_A12* controls fuzz fiber initiation, while both *GhMYB25like_A12* and *GhMYB25like_D12* regulate lint fiber development. Sequencing analyses showed that the *fbl*_SHZ_ mutant exhibited a K104M mutation in the R2R3 domain of *GhMYB25like_A12* and a transposable element insertion in *GhMYB25like_D12*, leading to fiberless seeds. Knockout of *GhMYB25like_A12* produced fuzzless seeds, knockout of *GhMYB25like_D12* led to no obvious change in seeds, and knockout of both (*GhMYB25like_A12*&*D12*) resulted in fiberless seeds. The 12:3:1 ratio reappeared in the F_2_ population developed from the *GhMYB25like_A12*&*D12* mutated plants as female and Jin668 as the male, which further confirmed the genetic interaction observed in *fbl_SHZ_*. RNA-seq analysis revealed that *GhMYB25like* regulates cotton fiber initiation through multiple pathways, especially fatty acid metabolism. This study elucidates the key genes and their genetic interaction mechanisms governing cotton fiber initiation, providing a theoretical foundation for genetic improvement of cotton fiber traits.

## Simple Summary

Cotton produces two distinct types of seed fibers: short fuzz and long lint, with lint fiber initiation being the primary determinant of cotton yield as the world’s most important natural textile source. However, the genetic regulation underlying fiber initiation exhibits considerable complexity. This study characterized the mutant loci responsible for the fiberless (fuzzless–lintless, seeds without both lint and fuzz) phenotype in a naturally fiberless cotton mutant (*fbl_SHZ_*), identifying duplicated *GhMYB25like* genes on homologous Chr A12 and Chr D12 as key regulators of both lint and fuzz fiber initiation. Genetic analysis revealed a dominant epistasis with the fuzz gene exerting dominance over the lint gene. Furthermore, the study demonstrated that these genes influence fiber initiation through multiple biological processes, particularly fatty acid metabolism. These findings provide fundamental insights into the genetic mechanisms governing cotton fiber initiation, offering valuable theoretical foundations for yield improvement in cotton breeding.

## 1. Introduction

Cotton fiber originates from the ovule epidermis, and most cultivated cotton varieties possess two types of fiber on the seed coat: the long spinnable lint fiber and the short fuzz that firmly adheres to the seed coat. Lint fiber initiates on or before the day of flowering, and fuzz fiber initiates at 4–5 days post-anthesis (DPA) [1]. The number of fiber cell initiations contributes to the lint percentage, a vital factor for cotton fiber yield. Identifying the genes involved in lint and fuzz initiation would provide insights into regulating cell patterning [2]. What is more, it is significant to breed varieties with higher yields of lint fiber in the future.

Cotton fiber initiation is complicated, and the genetics and molecular basis underlying lint and fuzz initiation remain totally uncharacterized. Fiber initiation shares a similar model of cell fate determination with Arabidopsis leaf trichomes in many ways [3,4]. Up to now, progress has been made in discovering genes regulating cotton fiber initiation. MYB-domain transcription factors (TFs) are vital in developing cotton fiber and leaf trichome [4,5,6]. The MYBMIXTA-Like (MML) TFs are crucial regulators of epidermal cell differentiation. Ten MYB-MIXTA-like homologs (*GhMMLs*) are all highly expressed during fiber initiation in cultivated cotton [6,7], which contains the typical protein motif AQWESARxxAExRLxRES [8].

Numerous studies have demonstrated that the MYB-MIXTA-like (MML) TFs are essential in cotton fiber development. *MML3* (*GhMYB25like*) is especially expressed in the ovule epidermis and probably acts most upstream of the fiber initiation regulatory networks. RNA interference suppression of *GhMYB25like* led to fiberless seeds but normal trichomes elsewhere, which showed that *GhMYB25like* specifically promoted the initiation of fiber cells but not trichomes on cotton plants [4]. The small interfering RNAs (siRNAs) from bidirectional transcripts of *GhMML3_A12* (*GhMYB25like_A12*) mediated self-cleavage, which caused extremely low expression of *GhMML3_A12* and a fuzzless phenotype in *N_1_* mutant [9]. Both sub-genome homologs of *GbMML3* (*GbMYB25like_A12* and *GbMYB25like_D12*) contributed to lint initiation, while only *GbMYB25like_D12* promoted fuzz formation in a recessive manner in *Gossypium barbadense* [10]. The *GhMML4_D12* was first reported as the lint fiber development gene (*Li_3_*); the downregulated expression of *GhMML4_D12* in *n_2_NSM* (fuzzless–linted) plants resulted in a significant reduction in epidermal cell prominence and lint fiber production, but did not result in the lintless phenotype [11]. Recently, it was shown that *GhMML3_D12* (*GhMYB25like_D12*) was also responsible for the *n_2_* loci in *n_2_NSM*. Overexpression of *GhMML3_D12* in *n_2_NSM* restored fuzz fiber development, while knockout of *GhMML3_D12* in wild-type cotton resulted in a fuzzless–linted phenotype [12]. *GhMML3_D12* was also reported as the candidate gene for the *li_3_* locus of *Xu142 fbl*, and a retrotransposon was found in the second exon of *GhMML3_D12*, which was considered to be the real underlying mutation for *li_3_* [13]. It could be shown that the *MML3* TFs played a significant role in cotton fiber initiation. Still, it was disputable whether the function of the MML3 was on fuzz and lint initiation. Recently, the developmental trajectory starting from early differentiated fiber cells was reconstructed by scRNA-seq on cotton ovules, which showed that the high expression of *GhMYB25like* lasted until 3 DPA, and *GhMYB25like* not only participated in lint initiation but also in fuzz initiation [14]. Subsequently, it was reported that the dominant negative mutation in the GhMYB25like_A12 protein exerted its dominant negative effect by suppressing the activity of the normal GhMYB25like protein to reduce the lint and fuzz initiation in our previous study [15]. Thus, these studies have indicated the positive function of *GhMYB25like* on cotton fiber initiation; even so, it is difficult to articulate how *GhMYB25like_A12* and *GhMYB25like_D12* mediated fuzz and lint initiation.

In 2013, a fiberless mutant was found in the field of Cotton Research Institute, Shihezi Academy of Agriculture Science, Shihezi, Xinjiang, China (44° N, 86° E), and was named *fbl_SHZ_*. In this study, we aim to (1) develop F_2_ segregation populations to uncover the genetic behavior of the fiberless trait; (2) identify the causal genes underlying this trait through map-based cloning approaches; and (3) functionally validate the roles of candidate genes through transgenic technology.

## 2. Materials and Methods

### 2.1. Plant Materials

After several rounds of selfing in Wuhan, Hubei province, China (30° N, 114° E), and in Sanya, Hainan, China (18° N, 109° E), the wild type (WT) and the *fbl_SHZ_* were planted in the field of Huazhong Agricultural University, Wuhan, in May 2015, and F_1_ was made by crossing WT as the female parent and *fbl_SHZ_* as the male parent. The F_1_ seeds were planted in Sanya, Hainan, in the winter, and were self-pollinated to develop an F_2_ mapping population. The F_2_ population, including 1848 individuals and their parents, was planted in the experimental field at Huazhong Agricultural University, Wuhan, in May 2016. Another larger F_2_ population with 3100 individuals was grown to fine-map the *fbl_SHZ_* genes in the experimental field at Huazhong Agricultural University, Wuhan, in May 2018.

### 2.2. Agricultural Traits Evaluation of the fbl_SHZ_

Twenty WT plants and twenty *fbl_SHZ_* plants were selected to evaluate the plant height, branch number, and effective bolls. The epidermal hair on the stem, petiole, leaf, and petals was also compared to the WT and *fbl_SHZ_* plants. The samples of 0 DPA ovules from WT and *fbl_SHZ_* were harvested to observe the fiber initiation.

### 2.3. Genetic Mapping of the fbl_SHZ_

The leaves of independent F_2_ plants were collected for DNA extraction via the modified CTAB method [16]. A total of 5152 SSR markers derived from a genetic map constructed from a BC_1_ population between *G. barbadense* acc. 3-79 and *G. hirsutum* cv. Emian22 was used to screen polymorphic markers [17]. The 102 recessive fiberless plants from the F_2_ populations planted in 2016 were used for the primary mapping of *fbl_SHZ_*. All 286 recessive fiberless plants from the F_2_ populations planted in 2016 and 2018 were used for fine mapping of *fbl_SHZ_*. The leaves of 30 individuals with fuzzy–linted plants and 30 individuals with fiberless plants were mixed, respectively, to build fuzzy–linted bulk and fiberless bulk from the F_2_ population in 2016 for the linkage markers screening. The two parents were sequenced by Illumina sequencing to exploit molecular markers to narrow down the mapping region. The clean sequencing reads were mapped to the *G. hirsutum* reference TM-1 genome [18] by the BWA v0.2.0 software [19], and the SNPs and InDels (Insertion/Deletion) from the sequenced data of WT and *fbl_SHZ_* were identified using the Genome Analysis Toolkit (GATK v4.3.0.0) software [20]. The SNPs located within the candidate region were used for Kompetitive Allele-Specific PCR (KASP) markers development, and the method of KASP markers development and genotyping followed our previous studies [21]. The genetic linkage analysis of the target *fbl_SHZ_* gene was performed by Mapmaker3.0, and the genetic map was obtained by the QTL IciMapping v4.1.0.0 software [22].

### 2.4. Gene Cloning, Vector Construction, and Cotton Transformation

The primers of the candidate genes were designed based on the TM-1 reference genome [18] by Primer Premier v5.00 software. The full length of *GhMYB25like_A12* and *GhMYB25like_D12* was amplified from WT and *fbl_SHZ_*, and ligated into the pGEM-T Easy cloning vector (Biotech Co. Ltd., Promega, Beijing, China); the positive clones were sequenced by Wuhan Tsingke Biotechnology Co., Ltd. (Wuhan, China). The final sequences were analyzed with DNAMAN v6 software. The CRISPR-Cas9-mediated gene editing vector for *GhMYB25like_D12* was constructed by infusion reactions, and the standard methodology was followed from the previous study [23]. The gene editing vector was then introduced into *G. hirsutum* acc. Jin668 by *Agrobacterium tumefaciens-mediated* transformation. Transgenic cotton lines were grown in the greenhouse (28–35 °C by day and 20–25 °C by night) under a 16/8 h light/dark cycle. All the primers used for vector construction are listed in Table S1.

### 2.5. On-Target Analysis of Gene-Edited Plants

The T_0_ transgenic plants were screened by PCR analysis using Cas9 primers (Table S1). High-throughput tracking of mutations (Hi-TOM) was performed to identify the mutated alleles in T_1_ transgenic lines [24]. Firstly, the targeted regions were amplified by PCR using site-specific primers. Then, barcode primers were used to add barcodes to the first-round PCR products. The products of all the samples were mixed in equal amounts and purified to perform next-generation sequencing (NGS). Last of all, the NGS data was analyzed using the Hi-Tom platform (http://hi-tom.net/hi-tom/ accessed on 28 July 2025).

### 2.6. Scanning Electron Microscopy (SEM)

Ovule samples were collected at 0 DPA from the WT, *fbl_SHZ_*, Jin668, and transgenic plants. The samples were immediately fixed in 2.5% (*v*/*v*) glutaraldehyde at 4 °C. Following fixation, they were dehydrated through a graded ethanol series, transferred to amyl acetate, and then dried to the critical point. Fiber initiation was observed and photographed with a JSM-6390/LV SEM (Jeol, Tokyo, Japan).

### 2.7. RNA Extraction and Real-Time Quantitative PCR (RT-qPCR)

All the samples used for RNA extraction were collected from the experimental field at 9–10 a.m. on a sunny day. The −3 DPA, −1 DPA, 0 DPA, +1 DPA, and +3 DPA ovules were harvested and immediately frozen in liquid nitrogen and then stored at −80 °C. Total RNA was extracted using the RNA prep Pure Plant Kit (TIANGEN Biotech, Beijing, China). A total of 3 μg RNA for each sample was reverse transcribed into cDNA using M-MLV reverse transcriptase (Promega). The cDNA was used as a template for RT-qPCR using the 7500 Real-Time PCR System (Applied Biosystems), and *GhUBQ7* (*Ghir_A11G011460*) was used as the internal reference. Three repetitions of each sample were applied. All the primers used for RT-qPCR are listed in Table S1.

### 2.8. Subcellular Localization

The full-length CDS of *GhMYB25like* was amplified from both WT and *fbl_SHZ_*, then independently inserted into the pGWB741 vectors with GFP fused to the N-terminal via Gateway BP and LR recombination reactions (Invitrogen, America). The 35S::GFP-*GhMYB25like* construct was transiently expressed in tobacco epidermal cells following *Agrobacterium*-mediated transfection. *Nicotiana benthamiana* was grown in 16/8 h light/dark conditions under white, fluorescent light at 25 °C. GFP fluorescence was detected under a confocal microscope (Olympus FV1200, Tokyo, Japan) after 48 h following *Agrobacterium* transfection. All the primers used for vector constructions are listed in Table S1.

### 2.9. Transcriptome Sequencing

The −3 DPA, 0 DPA, and +1 DPA ovules from Jin668 and Cr-*GhMYB25like_At&Dt* (*GhMYB25like_A12* and *GhMYB25like_D12* were both mutated) CRISPR-Cas9-edited lines were used for RNA extraction. RNA-seq was performed using the Illumina HiSeq 2000 system. The clean RNA-seq reads were mapped to the TM-1 reference genome [18] by HISAT version 2.0 [25]. FeatureCounts were used to calculate the transcript levels of annotated genes [26]. Differentially expressed genes (DEGs) were determined using the threshold of *p* ≤ 0.05 and the absolute value of the log_2_fold change ≥ 1.

## 3. Results

### 3.1. Phenotype and Inheritance of the Fiberless Mutant fbl_SHZ_

**Figure 1:**
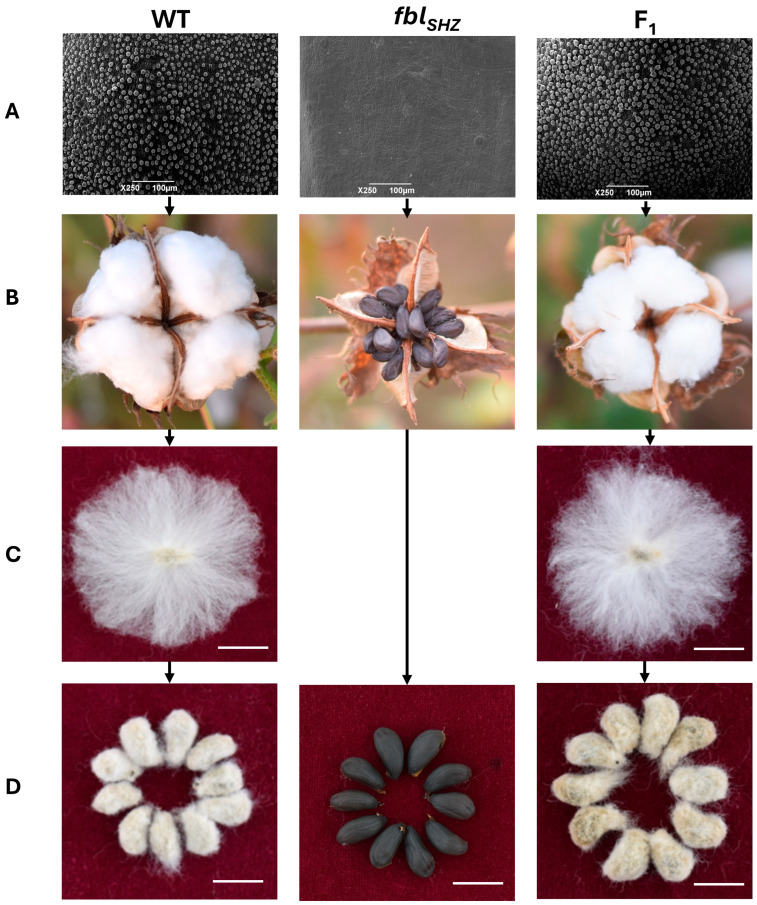
Phenotypic observation of cotton lint and fuzz fiber in WT, *fbl_SHZ_*, and F_1_: (**A**) The ovules at 0 DPA were observed by electron microscopy. Bars = 100 µm. (**B**) Phenotypic observation of three materials during boll opening. (**C**,**D**) Seed phenotype after combing and ginning of fibers. Bars = 1 cm.

To assess potential developmental differences, the plant height, branch number, and effective bolls were tested from 20 WT plants and 20 *fbl_SHZ_* plants; no statistically significant differences were found in these agronomic traits between WT and *fbl_SHZ_*. Moreover, the epidermal hair on the stem, petiole, leaf, and petal was also compared between the WT and *fbl_SHZ_* plants, and fewer trichomes were present in *fbl_SHZ_* than in WT (Figure S1). To investigate fiber cell initiation, scanning electron microscopy (SEM) was performed on 0 DPA ovule epidermis from the WT, *fbl_SHZ_*, and F_1_ hybrid plants. It revealed that the fiber cells of WT and F_1_ protuberated normally from the surface of the ovule, while no fiber cells protruded on the surface of the ovule in *fbl_SHZ_* (Figure 1).

To fine-map the *fbl_SHZ_* gene, two F_2_ mapping populations (*fbl_SHZ_* × WT) of 1848 and 3100 individuals were developed in 2016 and 2018, respectively. Three phenotypes appeared in the F_2_ populations (Figure S2), including 1428 fuzzy–linted plants, 318 less/no fuzzy but linted plants, and 102 fiberless plants in the population of 2016, and the segregation ratio was 12:3:1 (χ^2^ _12:3:1_ = 5.19); and 1900 fuzzy–linted plants, 550 less fuzzy but normal linted plants, and 184 fiberless plants in the population of 2018, which also occurred with 12:3:1 segregation ratio (χ^2^ _12:3:1_ = 3) (Table S2). These results showed that two loci conferred the fiberless trait in *fbl_SHZ_*, and the fuzz fiber was epistatic to the lint fiber.

### 3.2. Genetic Mapping of the fbl_SHZ_ Gene

**Figure 2:**
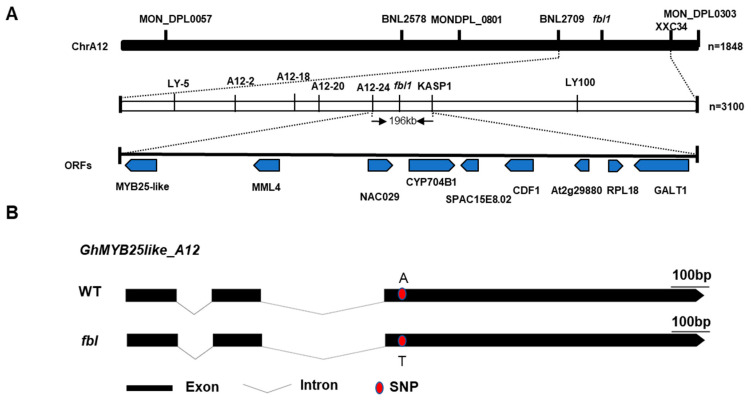
Map-based cloning of *fbl_SHZ_*: (**A**) Map-based cloning of the *fbl_SHZ_* locus. It was first mapped on Chr A12 between the markers BNL2709 and XXC34 using an F_2_ generation with 1848 individuals. It was further fine-mapped to a region between markers A12-24 and KASP1 using 3100 individuals. The mapping area was narrowed down to a 196 kb genomic interval, including nine predicted genes: *MYB25like*, *MML4*, *NAC029*, *CYP704B1*, *SPAC16E8.02*, *CDF1*, *At2g29880*, *RPL18*, and *GALT1*. (**B**) The gene structure of the *GhMYB25like_A12* between WT and *fbl_SHZ_*.

To primarily map the *fbl_SHZ_* gene, markers were selected at every 10 cM distance from the 5152 markers in the genetic map constructed by a BC_1_ population of *G. barbadense* acc. 3-79 and *G. hirsutum* cv. Emian22, and 30 polymorphic SSR markers between WT and *fbl_SHZ_* were screened out (Table S3). To identify markers linked with *fbl_SHZ_*, bulked segregant analysis was performed using the fuzzy–linted (WT) and fiberless (*fbl_SHZ_*) pools. A total of 102 recessive individuals from the F_2_ population in 2016 were used for genotyping and linkage analysis. Chi-square test of the linkage relationship between SSR markers and *fbl_SHZ_* indicated that the SSR markers from Chr A12, BNL2709, MON_DPL0303, BNL2578, MON_DPL0801, and MON_DPL0057 were linked to *fbl_SHZ_* (Table S3). Thus, *fbl_SHZ_* was anchored to Chr A12 between BNL2709 and MON_DPL0801 with genetic distances of 1.8 cM and 35.2 cM, respectively. To narrow down the interval further, 75 SSR markers between the region of BNL2709 and MON_DPL0801 were developed, and only the SSR marker XXC_34 was linked to *fbl_SHZ_,* with a genetic distance of 5.2 cM. Thus, the *fbl_SHZ_* locus was mapped between SSR markers BNL2709 and XXC_34 (Figure 2A). According to the sequence variations between WT and *fbl_SHZ_*, 100 InDel markers and 10 KASP markers in the candidate region were developed to narrow down the mapping region further, and 7 InDel markers and 1 KASP marker were polymorphic between the two parents. The mapping region was further narrowed down to a 196 kb region flanked by the Indel marker A12-24 and the KASP marker KASP1 using the 291 recessive individuals from the two F_2_ populations, with genetic distances of 1.03 cM and 0.86 cM, respectively (Figure 2A). Within this region, nine putative open reading frames (ORFs) were found, including the MYBMIXTA-like (MML) transcriptional factors, *GhMML3_A12*/*GhMYB25like_A12* [4,9] and *GhMML4_A12* (MYB106), which was the homology from the Dt genome (*GhMML4_D12*) [11]. The others were NAC transcription factor 29 (NAC29), Cytochrome P450 704B1 (CYP704B1), uncharacterized endoplasmic reticulum membrane protein C16E8.02 (SPAC16E8.02), cyclic dof factor 1 (CDF1), uncharacterized protein At2g29880 (At2g29880), 50S ribosomal protein L18 (RPL18), and beta-1,3-galactosyltransferase GALT1 (GALT1) (Table S4). Given that our previous study proved that *GhMYB25like_A12* is responsible for fuzz fiber development [15], and numerous studies have indicated that *GhMYB25like* acts as a hub in a regulatory network that controls both lint and fuzz initiation [4,9,12], it was identified as the candidate gene for *fbl_SHZ_*.

### 3.3. Cloning of the fbl_SHZ_ Gene

**Figure 3:**
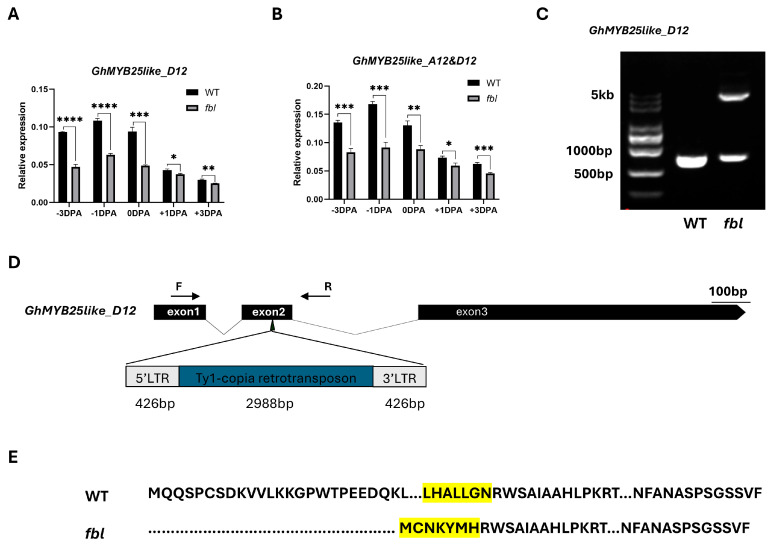
A TE insertion was observed in *GhMYB25like_Dt* in *fbl_SHZ_*. The relative expression of *GhMYB25like_D12* (**A**) and *GhMYB25like* (**B**) in the WT and *fbl_SHZ_* ovules during fiber initiation at −3, −1, 0, +1, +3 DPA, as determined by RT-qPCR. The *GhUBQ7* (*Ghir_A11G011460*) was used as the internal reference. Error bars represent ± SD. Significance was calculated using a *t*-test (**** *p* < 0.0001; *** *p* < 0.001; ** *p* < 0.01; * *p* < 0.05). (**C**) A retrotransposon insertion in the gene body of *GhMYB25like_D12* in *fbl_SHZ_*. The primers used for retrotransposon amplification were labeled by F and R, which were shown in (**D**). (**D**) Gene structure of the *GhMYB25like_D12* in WT and *fbl_SHZ_*. The retrotransposon was inserted in the third exon of *GhMYB25like_D12*. (**E**) The varieties of protein sequences between WT and *fbl_SHZ_*. The retrotransposon insertion in *GhMYB25like_D12* led to a new protein in *fbl_SHZ_*, and the highlight codon means the conserved regions between WT and *fbl_SHZ_*.

The full-length genomic sequences of the candidate gene *GhMYB25like_A12* and the homologs from the Dt sub-genomes *GhMYB25like_D12* were amplified from WT and *fbl_SHZ_*. One SNP located on the R2R3 domain in *GhMYB25like_A12* was found to cause one nonsynonymous mutation from A to T (Figure 2B), causing an amino acid mutation from lysine to methionine (K104M) (Figure S3A). As the highly homologous sequences between the At and Dt genomes, only eight amino acids changed between *GhMYB25like_A12* and *GhMYB25like_D12* in the reference genome (Figure S3B). Specific primers were designed to analyze the expression of *GhMYB25like_D12*, revealing significantly lower transcript levels in *fbl_SHZ_* compared with the WT plants during critical fiber initiation stages (Figure 3A); the expression analysis of both *GhMYB25like_A12* and *GhMYB25like_D12* using nonspecific primers demonstrated consistently reduced transcript accumulation in *fbl_SHZ_* (Figure 3B). The resequencing data of *GhMYB25like_D12* from WT and *fbl_SHZ_* were aligned visually, and an obvious gap was found in *fbl_SHZ_* (Figure S4A); the same result was found in transcript sequence data (Figure S4B). In addition, it displayed a longer PCR product than the expected length in *fbl_SHZ_* than in WT (Figure 3C). A 3840 bp insertion fragment was found in the second exon of *GhMYB25like_D12* in *fbl_SHZ_* by Sanger sequencing (Figure 3D). Further sequence analysis indicated that the insertion fragment was a putative Ty1/copia long terminal repeat (LTR) retrotransposon (named GhMYB25like_D12_TE hereafter). The components of a typical Ty1/copia element, such as LTRs, gag, and pol, could be found in *GhMYB25likeD12* in *fbl_SHZ_*. The retrotransposon consists of a 426 bp 5′-LTR, a 2988-bp internal region, and a 426 bp 3′-LTR (Figure 3D). Interestingly, the insertion of GhMYB25like_D12_TE generated a novel transcript in *fbl_SHZ_* (Figure 3E), encoding 286 amino acids in *fbl_SHZ_,* with the C-terminal 280 amino acids being the same as the amino acids in WT (Figure S5). Since retrotransposon insertions often interfere with transcriptional regulation, the aberrant transcript structure in *fbl_SHZ_* presumably underlies the downregulation of *GhMYB25like_D12* during fiber initiation.

In conclusion, the fiberless phenotype in the *fbl_SHZ_* mutant should be caused by the nonsynonymous mutation in the R2R3 domain of *GhMYB25like_A12* and a TE insertion in the second exon of *GhMYB25like_D12*, which may impair the DNA binding activation to targets and interrupt the gene function, respectively.

### 3.4. Functional Verification of GhMYB25like

**Figure 4:**
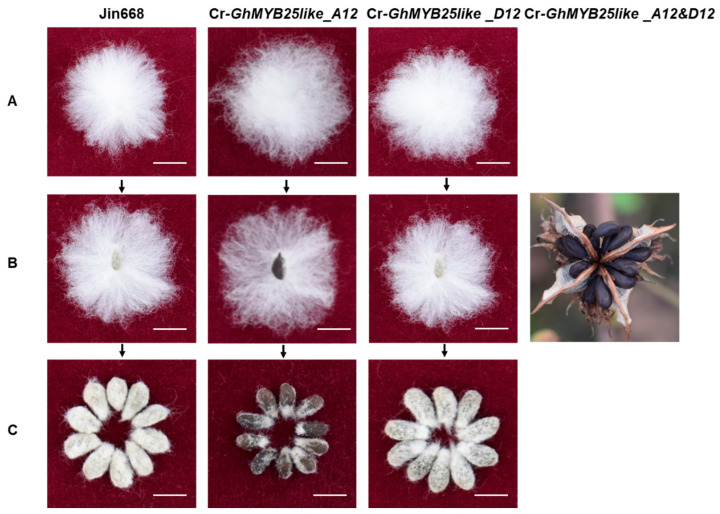
Phenotypic observation of cotton lint and fuzz fiber in *GhMYB25like* mutant lines: (**A**) Fiber phenotype in Jin668, *GhMYB25like_A12* mutant line, *GhMYB25like_D12* mutant line, and *GhMYB25like_A12&D12* (*GhMYB25like*) mutant line. Bars = 1 cm. (**B**) Seed phenotype after combing of fibers. Bars = 1 cm. (**C**) Seed phenotype after ginning of fibers. Bars = 1 cm.

To determine whether the amino acid variations in *GhMYB25like_A12* between WT and *fbl_SHZ_* affect protein localization, a subcellular localization assay was performed in *N. benthamiana* leaves, which showed that they were located in the cell nucleus and the variations did not influence the protein location (Figure S6). To comprehend the function of *GhMYB25like_A12* and *GhMYB25like_D12* on fiber initiation, specific sgRNAs with the CRISPR-Cas9 system were used to create mutant lines (the Cr_*GhMYB25like_A12* line was used in our previous study [15], and the Cr_*GhMYB25like_D12* line was generated in this study) (Figures S7 and S8). The transgenic plants showed that when *GhMYB25like_A12* was specifically knocked out (Cr-*GhMYB25like_A12* mutant line), no fuzz was found on the cotton seed coat compared with Jin668 (Figure 4); what is more, the trichome on the surface of the stem, petiole, leaf, and petal was reduced obviously (Figure S9); when *GhMYB25like_D12* was independently knocked out (Cr-*GhMYB25like_D12* mutant line), no significant difference was found on cotton seeds (Figure 4); it showed glabrous on cotton seed when both *GhMYB25like_A12* and *GhMYB25like_D12* were knocked out simultaneously (Cr-*GhMYB25like_A12&D12* mutant line), and no trichomes were covered on the surface of the stem, petiole, leaf, and petal (Figure S9).

### 3.5. The Fuzz Gene Was Dominant Epistatic to the Lint Gene

To uncover the phenotypes of the mutant lines further, an F_2_ segregation population was developed from a cross between the Cr-*GhMYB25like_A12&D12* mutant line as the female parent and Jin668 as the male parent. A total of 289 individuals were obtained, and the three phenotypes reappeared in the F_2_ populations, including 226 fuzzy–linted plants, 51 less/no fuzzy but linted plants, and 12 fiberless individuals; the segregation ratio was 12:3:1 (χ^2^ = 0.27) (Table S5), which revealed the dominant epistatic of fuzz gene to lint gene. The results showed that *GhMYB25like_A12* mainly regulated the fuzz initiation, while *GhMYB25like_A12* and *GhMYB25like_D12* were responsible for the lint fiber initiation.

### 3.6. GhMYB25like Is Involved in Regulating Fiber Initiation in Multiple Pathways

**Figure 5:**
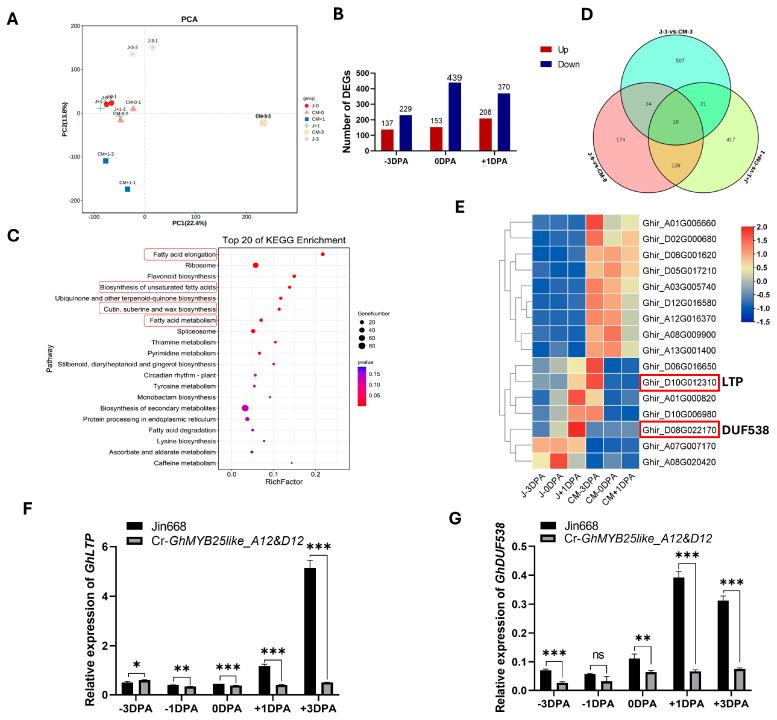
RNA-seq analysis of *GhMYB25like* mutant line during the fiber initiation at −3, 0, and +1 DPA ovules: (**A**) The PCA analysis. (**B**) The DEGs in three stages of fiber initiation in Cr-*GhMYB25like_A12&D12* mutant lines. (**C**) The top 20 of the KEGG enrichment analysis, and the red frames indicates the iterms of fatty acid related metabolism. (**D**) Venn diagram of the specific genes at the −3 DPA, 0 DPA, and +1 DPA ovule in the Jin668 and *GhMYB25like* mutant lines. (**E**) The heatmap of 16 DEGs in three stages. The relative expression of *GhLTP* (**F**) and *GhDUF538* (**G**) in the Jin668 and *GhMYB25like* mutant lines during fiber initiation at −3, −1, 0, +1, +3 DPA, as determined by RT-qPCR. The *GhUBQ7* (*Ghir_A11G011460*) was used as the internal reference. Error bars represent ± SD. Significance was calculated using a *t*-test (*** *p* < 0.001; ** *p* < 0.01; * *p* < 0.05; ns, no signifacance).

To understand the possible metabolic pathway and target genes of *GhMYB25like* during cotton fiber initiation, the −3 DPA, 0 DPA, and +1 DPA ovules from the Jin668 and Cr-*GhMYB25like_A12&D12* mutant lines were used for RNA-seq analysis, and PCA analysis is shown in Figure 5A. Based on the transcriptome data, 578, 366, and 592 differentially expressed genes (DEGs) were found in −3 DPA, 0 DPA, and +1 DPA, respectively (Figure 5B). Kyoto Encyclopedia of Genes and Genomes (KEGG) pathway enrichment analysis was used to categorize function annotations for the DEGs, and these DEGs were mainly enriched in fatty acid metabolism, flavonoid biosynthesis, cutin, suberine, and wax biosynthesis (Figure 5C), suggesting that a series of genes regulated networks through different pathways to initiate cotton fiber. Only 16 genes performed significant expression levels among all the DEGs during the three stages (Figure 5D), including the nonspecific lipid-transfer protein LTP and the unknown protein DUF538 (Figure 5E).

In this study, *GhLTP* exhibited extremely low expression levels in the Cr-*GhMYB25like_A12&D12* mutant line during cotton fiber initiation (Figure 5F), which revealed that *GhMYB25like* might target the *GhLTP* protein to regulate cotton fiber initiation directly or indirectly. The transcript level of *GhDUF538* was also sharply decreased in the Cr-*GhMYB25like_A12&D12* mutant line (Figure 5G), which implied its role in cotton fiber initiation.

## 4. Discussion

### 4.1. The Duplicated GhMYB25like Genes Regulate Cotton Lint and Fuzz Fiber Initiation

According to previous reports, *GhMYB25like* had been identified as the fuzz and lint loci in different fiber mutants through a map-based cloning method: the dominant fuzzless locus *N_1_* was controlled by the *GhMML3_A12* (*GhMYB25like_A12*) [9], the less mutant phenotype with lint and no fuzz mutant was induced by the dominant negative mutation *GhMYB25like_At*^hapT^ [15]; the lint fiber locus was indeterminate in different mutants, *GhMML4* and *GhMYB25like_D12* were considered as the genes that regulate the initial development of lint in fuzzless–linted mutant *n_2_NSM* [11,12]; *GhMYB25like_D12* was also reported as the *li_3_* in *Xu142 fl* mutant, a retrotransposon insertion in the second exon of *GhMYB25like_D12*, which decreased its expression and resulted in the lintless–fuzzless phenotype [13]. Here, a nonsynonymous mutation A/T with a K104M mutation in the R2R3 domain of *GhMYB25like_A12* was identified in the *fbl*_SHZ_ mutant, which was inconsistent with previous studies [4,9], while it was consistent with the variation in the recent study [15]. A retrotransposon insertion in the second exon of *GhMYB25like_D12*, which generated a novel but decreased transcription. The two variations may lead to the fiberless phenotype in the *fbl*_SHZ_ mutant.

In this study, *GhMYB25like_A12* and *GhMYB25like_D12* were identified as the fuzz and lint initiation genes in the *fbl_SHZ_* mutant. It presented fuzzless–linted, fuzzy–linted, and fiberless seeds when *GhMYB25like_A12*, *GhMYB25like_D12*, and *GhMYB25like_A12*&*D12* were knocked out by the CRISPR-Cas9 system, respectively. These results revealed that *GhMYB25like_A12* regulates fuzz initiation, while *GhMYB25like_A12* and *GhMYB25like_D12* coordinately control lint development. These results confirmed that *GhMYB25like* from At and Dt genomes played similar but different roles in cotton fuzz and lint initiation.

Our previous study elucidated the intricate functional mechanism of *GhMYB25like*, demonstrating that the mutant GhMYB25like protein acts as a dominant-negative regulator by inhibiting the activity of its wild-type counterpart during lint and fuzz initiation. Further investigation revealed that the degree of interference with normal GhMYB25like function correlated with the extent of R2R3 MYB binding domain preservation in the truncated mutant protein generated through gene editing. Specifically, mutant proteins retaining larger portions of the R2R3 domain exhibited stronger inhibitory effects on the activity of wild-type GhMYB25like, consequently more severely impairing fiber initiation [15]. The especially knocked out *GhMYB25like_D12* caused almost normal lint and fuzz fiber, which may be caused by no R2R3 domain preservation (Figure S8). A recent study demonstrated that the specific knockout of the *GhMYB25like_D12* locus resulted in fuzzless seeds, whose phenotypic effects were identical to those observed in the *GhMYB25like_A12* mutant line. Notably, the editing target was situated in the third exon of *GhMYB25like_D12*, yet the mutant protein retained an intact R2R3 DNA binding domain [12]. In summary, the regulation mechanism of *GhMYB25like_A12* and *GhMYB25like_D12* on cotton fuzz and lint initiation was intricate in the reported study.

### 4.2. The Dominant Epistasis of the Fiberless Mutant in Cotton

There are usually two or more copies of each gene located in the A and D sub-genome chromosomes in the *G. hirsutum* (AD)1 genome, representing homoeologous or duplicate copies of the diploid ancestral species A and D genomes [11]. *GhMYB25like_A12* shares high homology to *GhMYB25like_D12*, and there are only eight nonsynonymous differences between *GhMYB25like_A12* and *GhMYB25like_D12*. In the previous study, RNA interference suppression of *GhMYB25like* led to a fiberless phenotype, with no change in trichomes elsewhere [4]. On account of the high homology of *GhMYB25like* from At and Dt genomes, the fiberless seeds in RNAi transgenic cotton may be caused by the suppression of *GhMYB25like_A12* and *GhMYB25like_D12* simultaneously. In this study, knocking out *GhMYB25like* (both *GhMYB25like_A12* and *GhMYB25like_D12*) through the CRISPR-Cas9 system resulted in fiberless seeds and mostly glabrous elsewhere, compared with Jin668, and the phenotype of seeds was inconsistent with the previous study [4]. The especially knocked out *GhMYB25like_A12* caused fuzzless–linted seeds but normal trichomes elsewhere in our previous research [15]; it showed that *GhMYB25like_A12* was mainly responsible for fuzz production but not trichomes on plants elsewhere, which was consistent with previous studies [9]. The especially knocked out *GhMYB25like_D12* caused normal lint and fuzz fiber, resulting in no conspicuous change on the cotton plant elsewhere. In addition to this, the segregation ratio in the F_2_ segregation population developed by the cross of *GhMYB25like* mutant line and Jin668 was consistent with the result of the segregation ratio in the F_2_ segregation population, which was developed from a cross between WT and *fbl_SHZ_*. No fuzzy–lintless plants appeared in the F_2_ populations, which indicated that the fuzz gene was dominant epistatic to the lint gene, which was also proved by the previous research [11,12].

Based on current evidence, it was reasonable for us to speculate that two potential regulatory pathways for lint fiber initiation existed, with *GhMYB25like_A12* and *GhMYB25like_D12* serving as core transcriptional regulators. The observation that the targeted knockout of *GhMYB25like_D12* did not produce conspicuous phenotypic alterations in cotton seeds suggests potential functional compensation by *GhMYB25like_A12*. This compensatory mechanism may involve *GhMYB25like_A12* regulating downstream targets normally controlled by *GhMYB25like_D12*. In summary, the regulatory mechanism underlying fuzz and lint fiber initiation mediated by *GhMYB25like* homologs was intricate, and further investigation is needed.

### 4.3. GhMYB25like Regulates Cotton Fiber Initiation Through Multiple Pathways

As the key regulator in the regulatory network of fiber initiation, *GhMYB25like* regulates both lint and fuzz initiation by regulating the expression of various initiation-related genes. Some key TFs were identified in the downstream of *GhMYB25like*. The silencing of *GhMYB109*, homologous to Arabidopsis *GL1*, substantially reduced fiber production in transgenic cotton [2]. Silencing of *GhMYB25* caused a short fiber phenotype [2,3]. Silencing of *GhHD-1* resulted in retarded fiber initiation, and all three TFs located downstream of *GhMYB25like* in the TF-mediated networks of fiber initiation [27]. Only the silencing of *GhMYB25like* led to a fiberless phenotype [4], which showed the significant regulation of *GhMYB25like* on cotton fiber initiation.

According to the transcriptome analysis, *GhMYB25like* might regulate cotton fiber initiation through multiple pathways, with particularly significant involvement in fatty acid metabolism. In this study, two DEGs, nsLTP and DUF538, were further analyzed. The nonspecific lipid transfer proteins (nsLTPs) abundantly exist in plant tissues. The proteins are characterized by a tunnel-like hydrophobic cavity, which makes them suitable for binding and transporting various lipids [28]. They are deemed to function for the shuttling of lipids between membranes and across the cytoplasm and regulating the beta-oxidation of fatty acids in glyoxysomes and intracellular fatty acid pools [29]. The plant nsLTPs play a role in diverse activities, including cotton fiber development [30,31]. The DUF538 (domain of unknown function) protein family consists of proteins widely distributed in land plants but not in animals or yeasts [32,33]. It was reported that a pair of DUF538 proteins, SVB and SVBL, played roles in the transcriptional regulation of trichome development, modulating plant growth and trichome development through the transcriptional regulation of GL1, the key TF of trichome initiation in Arabidopsis [34]. One GhDUF538 protein was recently defined as a fiber cell cluster with the fiber marker genes (*GhMYB25*, *GhMML9*, and *GhHD1*) verified by situ hybridization assays [14]. The two genes downregulated significantly in *GhMYB25like* mutants during fiber initiation, suggesting they may function as direct or indirect downstream targets in the *GhMYB25like* mediated regulatory network controlling fiber initiation. To further elucidate this regulatory relationship, direct molecular validation approaches should be employed, including LUC reporter assays, Y1H (Yeast One-Hybrid), EMSA (Electrophoretic Mobility Shift Assay), or ChIP-qPCR (Chromatin Immunoprecipitation quantitative PCR).

## 5. Conclusions

This study demonstrated that the duplicated *GhMYB25like* genes (*GhMYB25like_A12* and *GhMYB25like_D12*) on homologous chromosomes coordinately regulate cotton fiber initiation, with *GhMYB25like_A12* primarily controlling fuzz initiation and both homologs regulating lint formation in the fiberless mutant *fbl_SHZ_*; the fuzz gene was dominant epistasis over the lint gene. A K104M mutation in *GhMYB25like_A12* and a TE insertion in *GhMYB25like_D12* collectively led to the fiberless seeds in *fbl_SHZ_*. *GhMYB25like* regulated fiber initiation primarily through modulation of fatty acid metabolic pathways. The study deciphers the laws of inheritance of fiberless mutants in cotton on the genetic level, advancing our understanding of the role of gene duplication in fiber initiation, which could guide us to develop different varieties of fuzz/lint to meet the multiple demands of the textile industry.

## Data Availability

The raw data used in this study can be accessed at Science Data Bank: https://www.scidb.cn/anonymous/bnVVajZi (accessed on 13 June 2025), https://www.scidb.cn/anonymous/VXp5QVZi (accessed on 13 June 2025), and https://www.scidb.cn/anonymous/WVZSalli (accessed on 13 June 2025).

## Supplementary Materials

The following supporting information can be downloaded at https://www.mdpi.com/article/10.3390/biology14080983/s1, Table S1. All primers were used in this study. Table S2. Phenotypic statistics of the F_2_ populations in the field. Table S3. Chi-Squared test of linkage relationship between SSRs and *fbl_SHZ_*. Table S4. The candidate genes in the *fbl_SHZ_* mapping locus. Table S5. Phenotypic statistics of the F_2_ population developed by the WT and *GhMYB25like_A12&D12* mutant line. Figure S1. Phenotypic observation of cotton plants in WT and *fbl_SHZ_*. Figure S2. Phenotypic observation of cotton lint and fuzz fiber in F_2_ populations. Figure S3. The protein sequence of GhMYB25like. Figure S4. The genome and transcript sequences visual alignment of *GhMYB25like_D12* in WT and *fbl_SHZ_*. Figure S5. The protein sequence alignment of GhMYB25like_D12 between WT and *fbl_SHZ_*. Figure S6. Subcellular localization assay of *GhMYB25like*. Figure S7. Characterization of cotton CRISPR mutant lines on *GhMYB25like*. Figure S8. The protein sequences of the GhMYB25like in CRISPR-Cas9 mutant lines. Figure S9. Phenotypic observation of cotton plants in *GhMYB25like* CRISPR mutant lines.

## Abbreviations

The following abbreviations are used in this manuscript:

CDS	Coding Sequence
CRISPR-Cas9	Clustered Regularly Interspaced Short Palindromic Repeptides-Cas9
CTAB	Hexadecyltrimethylammonium bromide
DEG	Differentially Expressed Gene
DPA	Days Post-Anthesis
GATK	Genome Analysis Toolkit
GFP	Green Fluorescent Protein
Hi-Tom	High-Throughput Tracking of Mutations
InDel	Insertion/Deletion
KASP	Kompetitive Allele-Specific PCR
KEGG	Kyoto Encyclopedia of Genes and Genomes
MML	MYB-MIXTA-like
NGS	Next-Generation Sequencing
PCR	Polymerase Chain Reaction
RNA-seq	RNA sequencing
RT-qPCR	Real-Time quantitative PCR
SEM	Scanning Electron Microscopy
SNP	Single Nucleotide Polymorphism
SSR	Simple Sequence Repeat
TE	Transposable Element
TF	Transcription Factor
WT	Wild Type
