# Correction: Le et al. Duplicated Genes on Homologous Chromosomes Decipher the Dominant Epistasis of the Fiberless Mutant in Cotton. *Biology* 2025, *14*, 983

**DOI:** 10.3390/biology14111481

**Published:** 2025-10-24

**Authors:** Yu Le, Xingchen Xiong, Zhiyong Xu, Meilin Chen, Yuanxue Li, Chao Fu, Chunyuan You, Zhongxu Lin

**Affiliations:** 1 National Key Laboratory of Crop Genetic Improvement, College of Plant Science and Technology, Huazhong Agricultural University, Wuhan 430070, China; leyu_hzau@163.com (Y.L.); xcxiong2016@163.com (X.X.); xuzhiyong@webmail.hzau.edu.cn (Z.X.); chenmeilin0623@163.com (M.C.); liyx1124@163.com (Y.L.); fuchao_666@webmail.hzau.edu.cn (C.F.); 2 Cotton Research Institute, Shihezi Academy of Agriculture Science, Shihezi 832011, China; 3 Xinjiang Uygur Autonomous Region Academy of Agricultural Sciences, Urumqi 830091, China

## Error in Figure

In the original publication [1], there was a mistake in Figure 4. Phenotypic observation of cotton lint and fuzz fiber in *GhMYB25like* mutant lines as published. There was an error in the image used for Jin668 in Figure 4B in the manuscript. The image for Cr-*GhMYB25like_D12* was mistakenly used in its place when organizing the figures. The correct image for Jin668 should be the one from the red-boxed area in the original image (Figure 1). Note: In the original image, ‘CMD’ refers to Cr-*GhMYB25like_D12* and ‘J668’ refers to Jin668.

The corrected **Figure 4. Phenotypic observation of cotton lint and fuzz fiber in *GhMYB25like* mutant lines** appears below (Figure 2).

The erroneous image for Jin668 in Figure 4B has been replaced with the correct one. The authors state that the scientific conclusions are unaffected. This correction was approved by the Academic Editor. The original publication has also been updated.

## Figures and Tables

**Figure 1:**
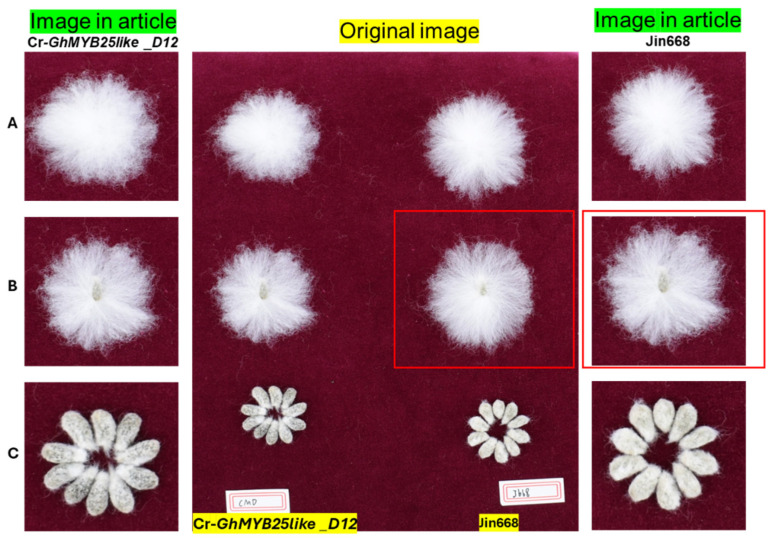
The error in Figure 4B.

**Figure 2:**
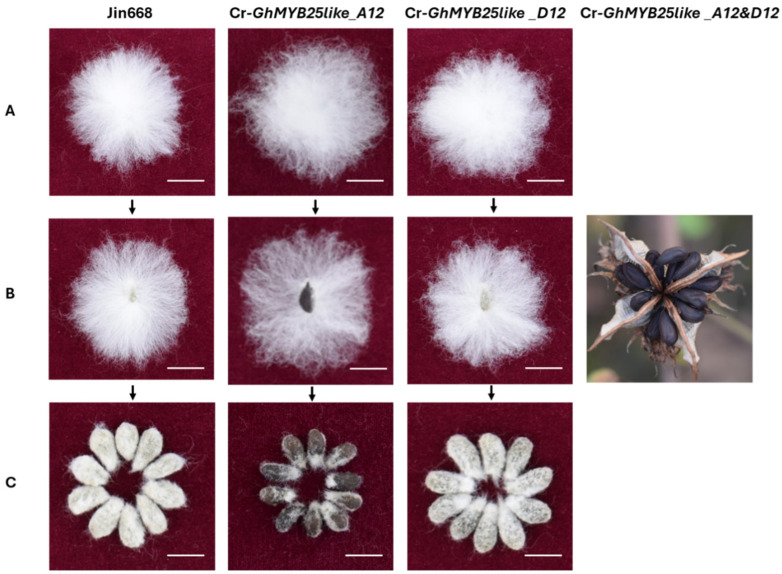
The corrected image for Figure 4B.
