# Supplementary material for: Duplicated Genes on Homologous Chromosomes Decipher the Dominant Epistasis of the Fiberless Mutant in Cotton
Source: Biology (Basel). 2025 Aug 2;14(8):983. doi: 10.3390/biology14080983 (PMC12383365; doi:10.3390/biology14080983)
Supplement: Supplementary file 1 [file biology-14-00983-s001.zip › Supplementary Figures.pdf]

Supplementary figures

Duplicated Genes on Homologous Chromosomes Decipher the Dominant Epistasis of the Fiberless Mutant in Cotton

Yu Le [1], Xingchen Xiong [1], Zhiyong Xu [1], Meilin Chen [1], Yuanxue Li [1], Chao Fu [1], Chunyuan You [2,*] and Zhongxu Lin [1,3,*]

1 National Key Laboratory of Crop Genetic Improvement, College of Plant Science and Technology, Huazhong Agricultural University, Wuhan 430070, China; leyu_hzau@163.com (Y.L.); xcxiong2016@163.com (X.X.); xuzhiyong@webmail.hzau.edu.cn (Z.X.); chenmeilin0623@163.com (M.C.); liyx1124@163.com (Y.L.); fuchao_666@webmail.hzau.edu.cn (C.F.)

2 Cotton Research Institute, Shihezi Academy of Agriculture Science, Shihezi 832011, China

3 Xinjiang Uygur Autonomous Region Academy of Agricultural Sciences, Urumqi 830091, China

* Correspondence: chunyuanyou@mail.hzau.edu.cn (C.Y.); linzhongxu@mail.hzau.edu.cn (Z.L.)

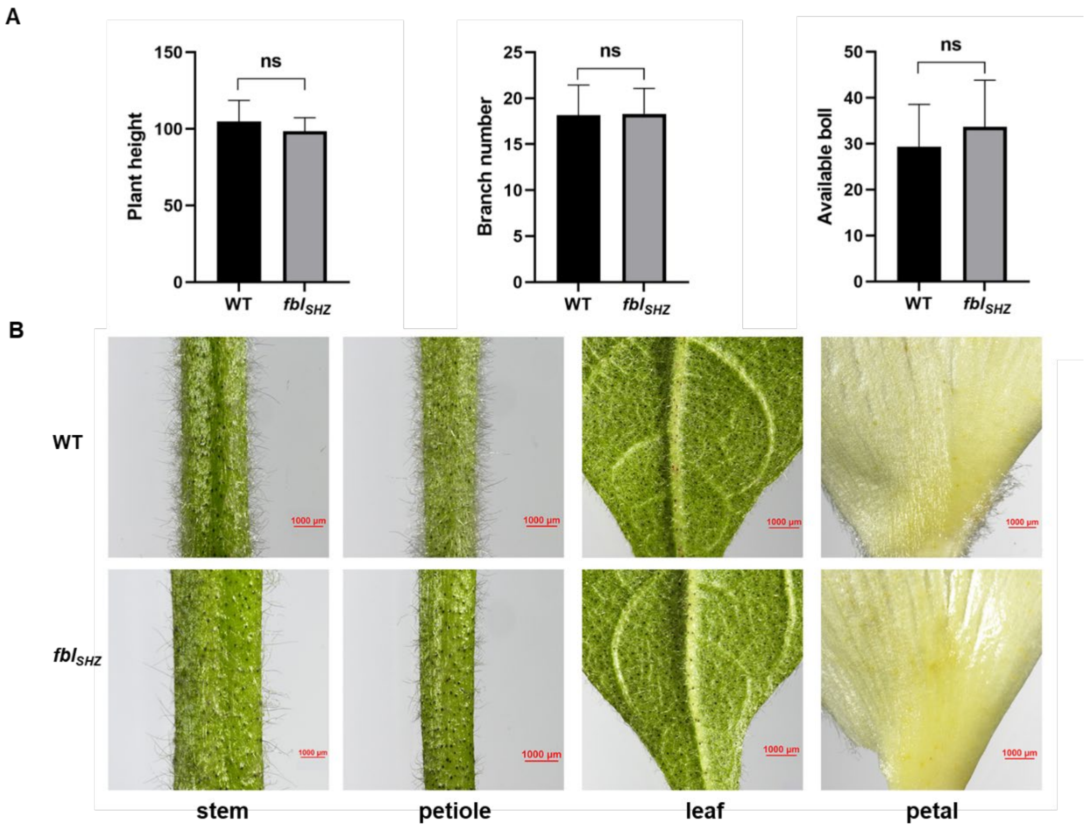

Figure S1. Phenotypic observation of cotton plants in WT and *fbl*$_{SHZ}$. (**A**) Observation of plant height, branch number, and available boll between WT and *fbl*$_{SHZ}$. (**B**) The epidermal hair observation on the stem, petiole, leaf, and petal in WT and *fbl*$_{SHZ}$. Bars = 1000 µm.

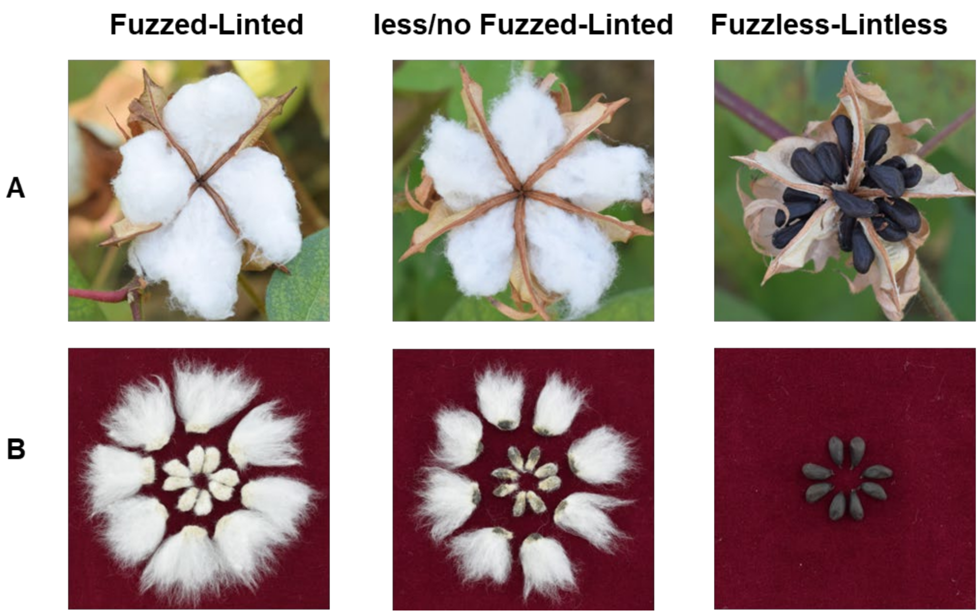

Figure S2. Phenotypic observation of cotton lint and fuzz fiber in F$_2$ populations. (**A**) The cotton bolls on the plants. (**B**) The phenotype of seeds after the combing of fibers. Three kinds of phenotypes were identified, which contained fuzzed-linted (with both fuzz and lint fiber on cottonseeds), less or no fuzz but linted fiber, fibreless (with either fuzz or lint fiber on cottonseeds).

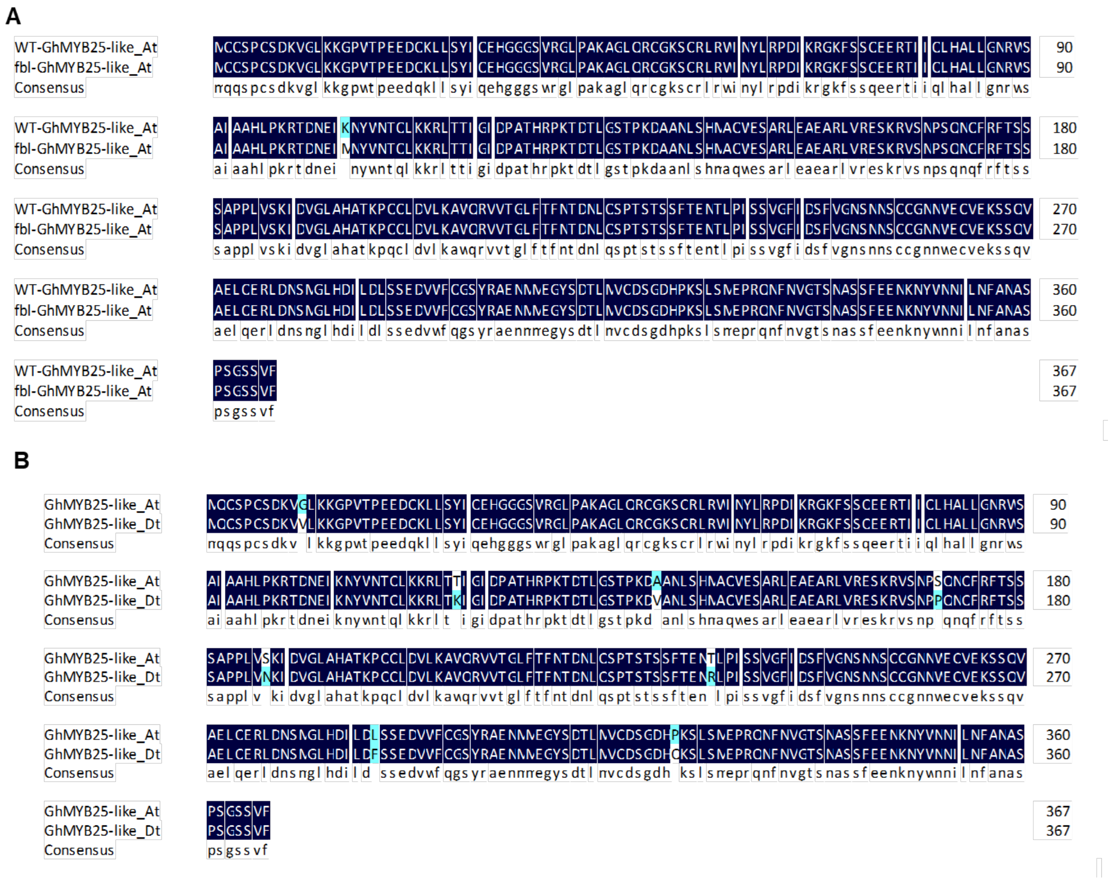

Figure S3. The protein sequence of GhMYB25like. (**A**) Protein sequence alignment of GhMYB25like_A12 between WT and *fbl*$_{SHZ}$. (**B**) Protein sequence alignment between GhMYB25like_A12 and GhMYB25like_D12 in TM-1.

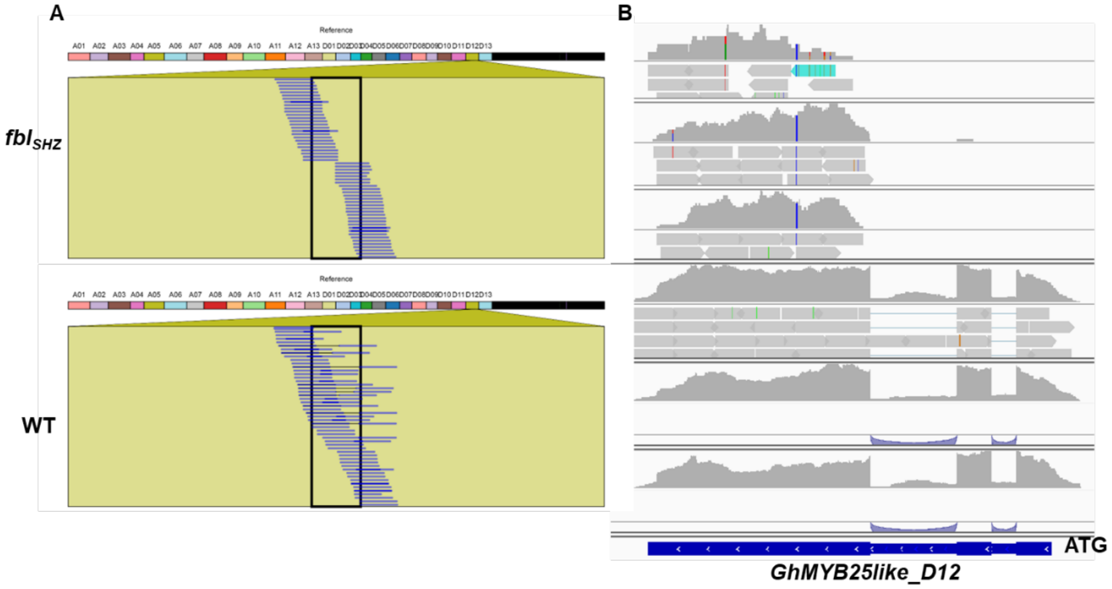

Figure S4. The genome and transcript sequences visual alignment of *GhMYB25like_D12* in WT and *fbl*$_{SHZ}$. (**A**) The visual sequences alignment of the genome of *GhMYB25like_D12* in WT and *fbl*$_{SHZ}$. An abruption was found in *fbl*$_{SHZ}$. (**B**) The visual sequences alignment of the transcript of *GhMYB25like_D12* in WT and *fbl*$_{SHZ}$. An abruption was found at *GhMYB25like_D12* in *fbl*$_{SHZ}$.

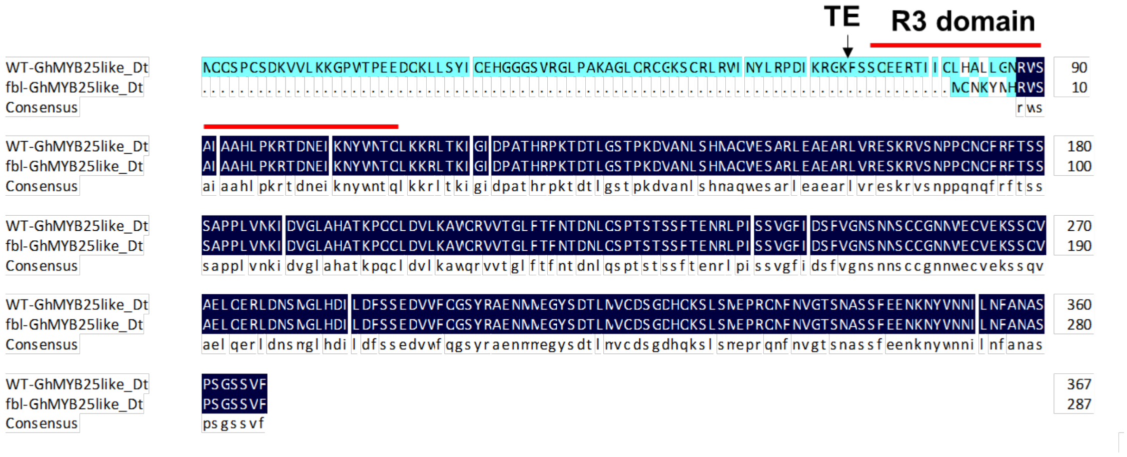

Figure S5. The protein sequence alignment of GhMYB25like_D12 between WT and *fbl*$_{SHZ}$. In *fbl*$_{SHZ}$, the TE was inserted between amino acids K and F, which was pointed out with a black arrow, and a new protein with 287 amino acids was generated; the red line indicated the R3 MYB domain.

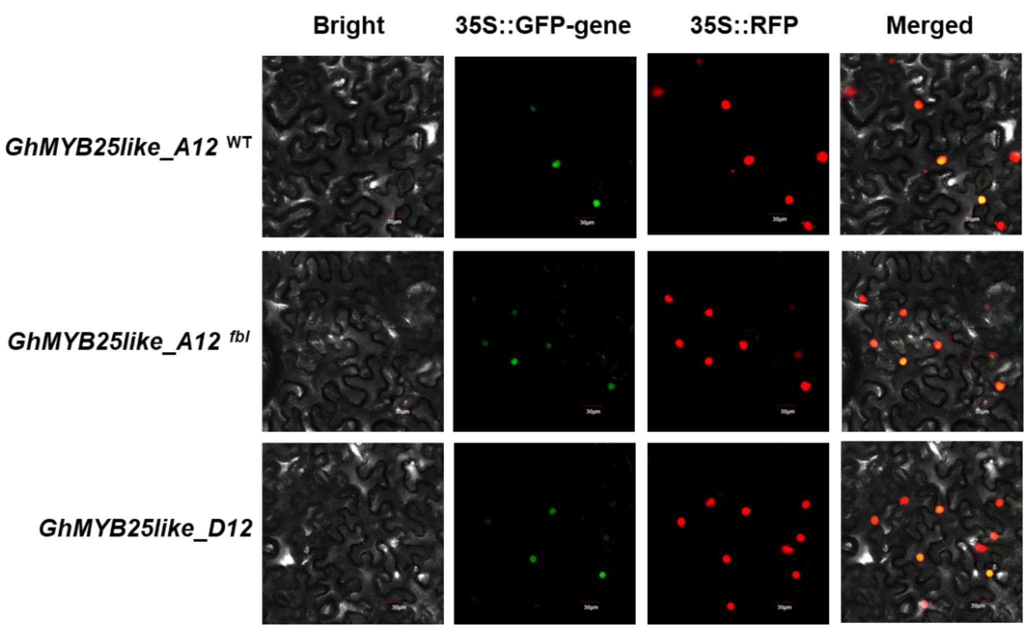

Figure S6. Subcellular localization assay of *GhMYB25like*. Subcellular localization of *GhMYB25like_A12*^{WT}, *GhMYB25like_A12*^{fblSHZ}, and *GhMYB25like_D12* protein in *N. benthamiana* leaves, 35S::GFP-gene represents the overexpression vector of *GhMYB25like* fused with GFP. The red fluorescent protein (RFP) was the marker in the cell nucleus, bars = 30 μm.

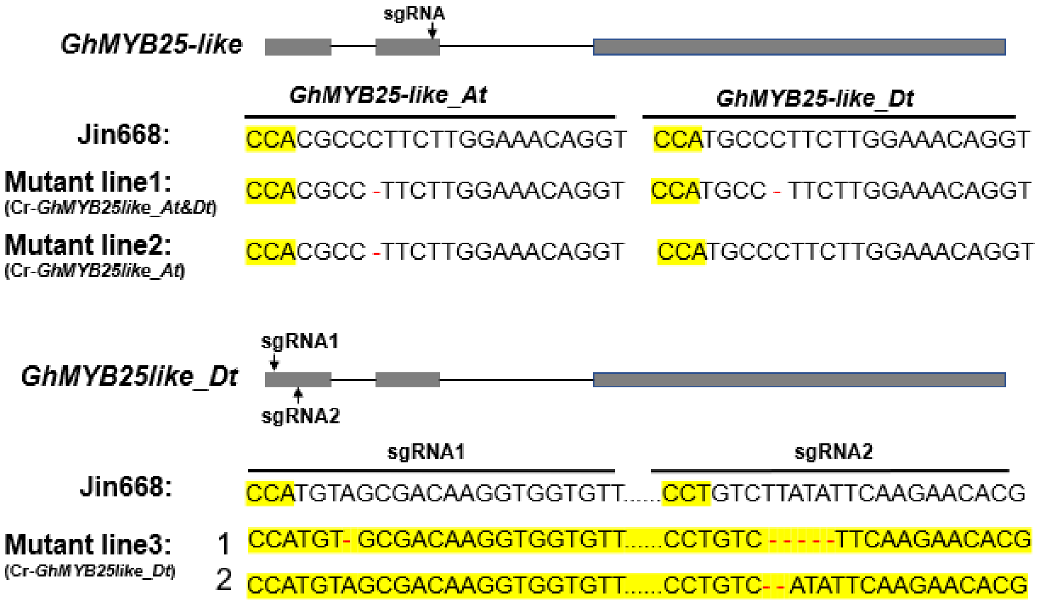

Figure S7. Characterization of cotton CRISPR mutant lines on *GhMYB25like*. The mutant line1 (Cr-*GhMYB25like_A12&D12*) represents the two homologous copies of *GhMYB25like* from Chr A12 and Chr D12 were mutated simultaneously, the mutant line2 (Cr-*GhMYB25like_A12*) represents the *GhMYB25like_A12* mutant, which is used

from our previous study (Zhao, et al, 2024), the mutant line3 (Cr-*GhMYB25like_D12*) represents the *GhMYB25like_D12* mutant line.

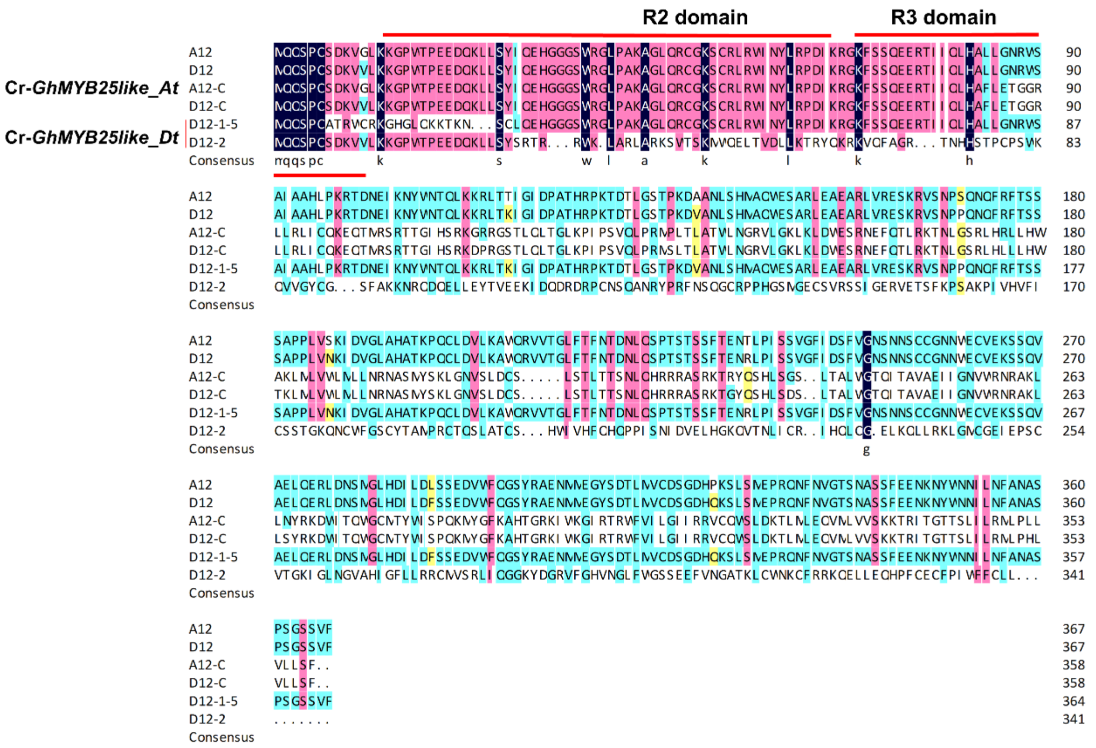

Figure S8. The protein sequences of the GhMYB25like in CRISPR/Cas9 mutant lines. A12 and D12 indicated the reference protein sequence of *GhMYB25like_A12* and *GhMYB25like_D12*; A12-C represents Cr-*GhMYB25like_A12*; D12-1-5 and D12-2 represent the two mutants in Cr-*GhMYB25like_D12*.

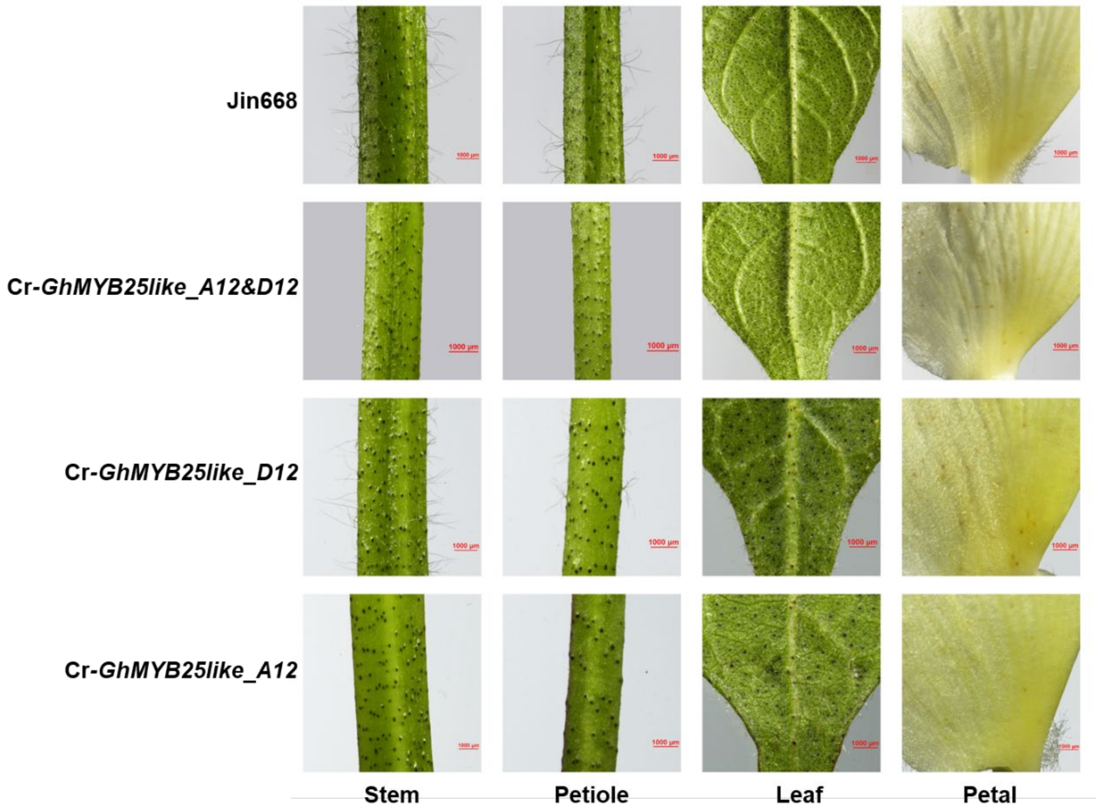

Figure S9. Phenotypic Observation of cotton plants in *GhMYB25like* CRISPR mutant lines. The epidermal hair observation on stem, petiole, leaf, and petal in Jin668, Cr-*GhMYB25like_A12&D12*, Cr-*GhMYB25like_D12*, and Cr-*GhMYB25like_A12* mutant lines. Bars = 1000 μm.
